# Poor Adherence to the WHO Guidelines on Feeding Practices Increases the Risk for Respiratory Infections in Surinamese Preschool Children

**DOI:** 10.3390/ijerph182010739

**Published:** 2021-10-13

**Authors:** Jill R. Wormer, Arti Shankar, Michael Boele Van Hensbroek, Ashna D. Hindori-Mohangoo, Hannah Covert, Maureen Y. Lichtveld, Wilco C. W. R. Zijlmans

**Affiliations:** 1 Department of Pediatrics, Elisabeth-TweeSteden Hospital (ETZ), 5022 GC Tilburg, The Netherlands; 2 Department of Biostatistics and Data Science, Tulane University School of Public Health and Tropical Medicine, New Orleans, LA 70112, USA; sarti@tulane.edu; 3 Amsterdam Centre for Global Child Health, Emma Children’s Hospital and Department of Global Health, Academic Medical Centre, University of Amsterdam, 1100 DD Amsterdam, The Netherlands; m.boele@amc.uva.nl; 4 Foundation for Perinatal Interventions and Research in Suriname (Perisur), Paramaribo, Suriname; ashna.mohangoo@perisur.org (A.D.H.-M.); wilco.zijlmans@uvs.edu (W.C.W.R.Z.); 5 Graduate School of Public Health, University of Pittsburgh, Pittsburgh, PA 15261, USA; hcovert@pitt.edu (H.C.); mlichtve@pitt.edu (M.Y.L.); 6 Faculty of Medical Sciences, Pediatrics, Anton de Kom University of Suriname, Paramaribo, Suriname

**Keywords:** respiratory tract infections, WHO infant and young child feeding indicators, Suriname

## Abstract

Poor feeding practices in infants and young children may lead to malnutrition, which, in turn, is associated with an increased risk of infectious diseases, such as respiratory tract infections (RTIs), a leading cause of under-five mortality. We explored the association between RTIs and the WHO infant and young child feeding (IYCF) indicators: minimum dietary diversity (MDD), minimum meal frequency (MMF), and minimum acceptable diet (MAD), among infants and preschool children in Suriname. A validated pediatric food frequency questionnaire was used and data on RTIs, defined as clinical care for fever with respiratory symptoms, bronchitis, or pneumonia were obtained. Associations between feeding indicators and RTIs were explored using hierarchical logistic regression. Of 763 children aged 10–33 months, 51.7% achieved the MDD, 88.5% the MMF, and 46.5% the MAD. Furthermore, 73% of all children experienced at least one upper and/or lower RTI. Children meeting the MDD and MAD had significantly lower odds on RTIs (OR 0.53; 95%CI: 0.37–0.74, *p* < 0.001; OR 0.55; 95%CI: 0.39–0.78, *p* < 0.001, respectively). The covariates parity and household income were independently associated with RTIs. In conclusion, MDD and MAD were associated with (upper) RTIs. Whether these indicators can be used as predictors for increased risk for RTIs should be assessed in future prospective studies.

## 1. Introduction

Significant progress has been made in reducing under-five mortality. Globally, the mortality rate declined from 93 deaths per 1000 live births in 1990 to 38 deaths in 2019 [1]. In Suriname, a middle-income country in South America, the under-five mortality rate was estimated at 18 deaths per 1000 live births in 2019, which is higher than the Latin American and the Caribbean average [2]. Despite the progress that has been made in reducing under-five mortality, the global burden of under-five deaths remains unacceptably high [1]. 

Acute RTIs are ranked among the leading causes of under-five mortality in developing countries. In 2013, the WHO estimated 9% of all under-five deaths in Suriname to be attributable to RTIs [3]. Numerous studies revealed striking intercountry and regional disparities in the occurrence of acute RTIs, and child survival outcomes associated with acute RTIs [4,5,6]. Mortality rates due to acute RTIs are 2–6 times higher in developing countries, as compared with developed countries [4,7]. The causes of these disparities are rooted in a complex interplay of socioeconomic and sociodemographic characteristics such as access to and quality of basic healthcare, household income level, exposure to environmental health hazards, as well as nutritional factors [4,8,9,10].

Studies have revealed an increased prevalence and mortality risk of infectious diseases in malnourished children [11,12,13,14,15]. Malnutrition is known to impair immunocompetence, causing increased susceptibility to infectious diseases. In turn, infections may deteriorate nutritional status through the loss of critical body stores and reduced intake [16,17]. 

Although Suriname performs relatively well against developing countries, the burden of malnutrition remains high. The latest national prevalence of under-five stunting, wasting, and overweight was 8.8%, 5.8%, and 4%, respectively, with the highest observed prevalences of these childhood adverse health conditions among those living in the interior [18]. Thus, nutritional interventions in Suriname harness great potential to indirectly reduce morbidity and mortality related to RTIs.

In 2008, the World Health Organization (WHO) and partners published a comprehensive set of valid indicators to serve as the benchmark for assessing infant and young child feeding (IYCF) practices. The indicators can be derived from population-based survey data and offer the advantage of inter-country and subnational comparisons of feeding practices. The key indicators reflecting dietary quality and quantity of complementary feeding are the minimum diversity diet (MDD), minimum meal frequency (MMF), and minimum acceptable diet (MAD) [19].

The Suriname Multiple Indicator Cluster Survey Six (MICS 6), implemented in 2018, made a significant effort to fill the data gap on the IYCF indicators [20]. To monitor progress made toward national and global Sustainable Development Goals (SDGs), harnessing comparative data was urgently needed. Additionally, to our knowledge, the association between feeding practices and respiratory tract infections has not been previously examined in Suriname. Understanding the associations between feeding practices and RTIs is essential for the development and implementation of effective interventions to reduce acute RTI-related morbidity and mortality. Therefore, the current country-specific study may be beneficial to health practitioners and IYCF policymakers.

Accordingly, the aim of this study was to determine the association between respiratory tract infections and the WHO infant and young child feeding (IYCF) indicators among infants aged 10–33 months in Suriname. Additionally, we aimed to determine and compare the proportion of infants meeting the indicators in three differently populated regions in Suriname where the study population resides of a larger CCREOH environmental study [21].

## 2. Materials and Methods

### 2.1. Study Design and Study Population

This research was embedded in the Caribbean Consortium for Research in Environmental and Occupational Health (CCREOH)-MeKiTamara study, an ongoing prospective longitudinal environmental epidemiologic cohort study of mother–child dyads in Suriname. This National Institutes of Health (NIH)-funded study (U01TW010087 and U2RTW010104) is designed to determine the potential impact of environmental exposures, including nutrition, on the health of pregnant women and on the early neurodevelopment outcomes of their subsequent offspring in the Surinamese regions of Paramaribo, Nickerie, and the tropical rainforest interior. The CCREOH-MeKiTamara study provides longitudinal follow-up of children from birth to 48 months. Data analyzed here were collected at the children’s 12-month follow-up. Details on study design, participants, demography, and follow-up were previously described [21].

Recruitment for the overall MeKiTamara study occurred in the first or second trimester of pregnancy at all hospitals and clinics of the Regional Health Services and the Medical Mission Primary Health Care Suriname. To be eligible to participate, respondents had to be aged 16 years or older, having a singleton pregnancy, planning to deliver at one of the participating hospitals and clinics, able to speak Dutch, or one of the local languages Sranan Tongo, Sarnami, Saramaccan, or Trio. 

From December 2016 to July 2019, a total of 1143 pregnant women were enrolled in the MekiTamara study. Subsequently, 77 women were excluded due to pregnancy termination, miscarriage, multiple gestations, consent withdrawal, and loss of contact, leaving 1069 participants. Ultimately, 1037 live births were assessed. Of the infants enrolled in the overall study, subjects were excluded for the following reasons: born < 33 completed weeks of gestation and/or had a birthweight < 2000 g, a significant medical or neurological condition—cerebral palsy, hydrocephalus, Down syndrome, or a significant visual or hearing impairment inconsistent with neurocognitive testing. These participants were excluded because of their potential effect on the neurodevelopment outcomes. Since data from the MeKiTamara study were used, these participants were also excluded from the current study. Ultimately, 799 children were eligible for data collection at 12 months of age.

### 2.2. Feeding Indicators

At the 12-month neurodevelopment follow-up visit, data were obtained in face-to-face interviews with enrolled mothers on their infants’ feeding practices using a Generation Suriname dietary questionary, modified from the validated Generation R year one questionnaire (with permission) and is available upon request [21,22]. 

In 2008, the WHO and partners proposed an updated set of core indicators to assess infant and young child feeding (IYCF) practices on an average day in children aged six (≥180 days) up to 24 months (<730 days) for the use in population-based surveys. The three specific IYCF indicators of interest concerning complementary feeding practices were the minimum dietary diversity (MDD), minimum meal frequency (MMF), and minimum acceptable diet (MAD) [19,23]. The data collected with the dietary Generation Suriname questionnaire were used to calculate the WHO indicators. These indicators were used to assess the feeding practices of all study children, including those older than 23 months (≥730 days). Infants above two years of age were included to increase the power of the study. 

The Generation Suriname dietary questionnaire did not distinguish vitamin-A-rich fruits and vegetables from non-vitamin-A-rich fruits and vegetables. Therefore, these had to be combined into one food group. Thus, our study had a total of six food groups instead of seven food groups: (1) grain roots and tubers, (2) legumes and nuts, (3) dairy products, (4) flesh foods, (5) eggs, and (6) vitamin-rich fruits and vegetables. For the children aged above two years, the following modifications were made to the indicators:

1. MDD: this indicator was considered achieved when receiving foods from at least four out of the six food groups [24];
2. MMF: the minimum requirement was defined as four times per day for breastfed infants and five times (three meals with two additional snacks) per day for non-breastfed children aged ≥ 24 months [25];
3. MAD: this composite indicator was considered achieved when the MDD and MMF indicators were met.

The modifications made to assess the feeding practices among the children aged ≥ 24 months, as well as combining two food groups, were consistent with the previously published study of Do et al. (2018) [24]. The details of the indicators and the modifications are available in Supplementary Table S1.

### 2.3. Infant Characteristics

Information regarding child characteristics such as gender, gestational age, being breastfed, food allergies, and age was obtained through the Generation Suriname questionnaire at the 12-month follow-up. Gender was categorized into male and female. Gestational age was determined based on the last menstrual period or early prenatal ultrasound (<16 weeks), if available. It was dichotomized into moderately preterm (33^+0^–36^+6^ weeks) and term births (37^+0^ weeks). Breastfeeding status was dichotomized into currently being breastfed or not being breastfed. Food allergies were parent-reported and dichotomized into a food allergy vs. no food allergy. Age in months was calculated by subtracting the birth date from the test date. It was explored as a continuous variable [21].

### 2.4. Maternal Demographics

Information on maternal sociodemographic and socioeconomic characteristics such as age, parity, ethnicity, marital status, educational level, and household income in Surinamese dollars (SRD) place of residence, timing of first antenatal care visit, and health insurance was obtained through various questionnaires at prenatal intake. 

Maternal age was calculated from the reported birthdate and explored as a categorical variable (16–19 vs. 20–24 vs. 25–29 vs. 30–34 vs. 35–39 vs. 40+ years). Parity was assessed with questions on reproductive history, including outcomes (abortions, miscarriages, live births, stillbirths). It was defined based on the number of previous live births and dichotomized into 0 vs. 1 previous live birth(s). 

Marital status, educational level, household income in SRD, place of residence, ethnicity, trimester for first antenatal care visit were self-reported. Marital status was dichotomized into married or living with a partner vs. not married or not living with a partner. Highest completed educational level was categorized into four subgroups (not educated/primary level vs. lower vocational/secondary vs. upper vocational/secondary vs. tertiary). Household income level was analyzed in eight subgroups (<400 vs. 400–799 vs. 800–1499 vs.1500–2999 vs. 3000–4999 vs. 5000–9999 vs. 10,000–14,999 vs. 150,000+ SRD).

Place of residence was categorized into three subgroups: (1) Paramaribo, Wanica; (2) Commewijne, Saramacca, Coronie, Nickerie, and Para; (3) tropical rainforest interior: Marowijne Brokopondo, and Sipaliwini. Participants were stratified into the following ethnic groups: Creole, Hindustani, Indigenous, Javanese, Tribal and mixed. Health insurance was dichotomized into insured vs. uninsured. First antenatal care visits were explored as categorical (first trimester vs. second trimester vs. third trimester) [21].

### 2.5. Respiratory Tract Infection

The Generation Suriname questionnaire was used to obtain information about upper (URTI) and lower respiratory tract infections (LRTI) [26,27,28]. Upper respiratory tract infections were defined by at least one parent-reported physician visit for serious colds, ear infections, or throat infections in the six months before the first follow-up. Lower respiratory tract infections were defined as at least one parent-reported physician visit for pneumonia, bronchitis, and bronchiolitis. Data concerning the severity or frequency of these respiratory tract infections were not collected.

### 2.6. Statistical Analyses

All statistical analyses were performed using IBM SPSS statistics for MacBook, version 27.0 (IBM Corp, Armonk, NY, USA). Prior to conducting statistical analyses, data were checked for completeness and consistency and cleaned. Cases with missing values were deleted from the dataset. Descriptive statistics were used to present baseline characteristics of enrolled participants. Categorical data were reported as frequencies and percentages (%), while continuous variables were presented as mean ± standard deviation (SD) when normally distributed and as medians and interquartile range (IQR) when not normally distributed. Independent samples *t*-test and chi-squared test were performed to compare continuous and categorical data, respectively, among the different age groups (<24 months and ≥24 months). Fisher’s exact test was used when 20% of the cells of a contingency table had an expected count below five. Hierarchical binary logistic regression models were constructed to determine the association between feeding practices and respiratory tract infections.

### 2.7. IYCF Indicators

The IYCF Indicators were expressed as dichotomous variables: value 0 for not achieving the indicator criteria and value 1 for achieving the criteria. The proportion of children meeting and not meeting the MDD, MMF, and MAD was calculated for children <24 months and ≥24 months of age. A multiple proportion test was conducted to calculate if the proportion of children meeting the indicators differed statistically across the three different regions: the capital Paramaribo, the semi-rural agricultural area of Nickerie in western Suriname, and the remote tropical rainforest interior. Additionally, a two-sample proportion test was performed to assess the differences in MAD between breastfed and non-breastfed children. Data in Table 4 were expressed as frequencies (N) and percentages (%).

### 2.8. Associations between Feeding Practices and Respiratory Health Effects

The exposure variables were the indicators MDD, MMF, and MAD (achieved vs. non-achieved). Information on URTI and LRTI was parent reported; therefore, it could have led to misclassification bias. Parent-reported diagnoses are less accurate outcome diagnoses than physician diagnoses. This possibility was considered by creating and analyzing a third subgroup, RTI, without distinguishing infections of the upper and lower tract. Therefore, three outcome variables were created: URTI, LRTI, and RTI.

The proportion of children with at least one parent-reported URTI (serious colds, ear infections, or throat infections), LRTI (pneumonia, bronchitis, or bronchiolitis), or RTI (serious colds, ear infections, throat infections, pneumonia, bronchitis, or bronchiolitis) was calculated for the achieved and non-achieved groups of the indicators. 

Two-sample test of proportions were conducted to calculate if the proportion of children with URTI, LRTI, or RTI differed statistically significant between achieving and not achieving the indicators. Next, binary hierarchical logistic regressions with a pre-specified order of inclusion for the blocks were used to develop predictive models for each of the the outcome variables (URTI, LRTI, and RTI).

The increment R^2^ at each step was taken as the component of variation. The regression models were cumulatively adjusted for the following covariates:

1. Currently breastfed;
2. Child characteristics: birth weight, gender;
3. Food allergies;
4. Maternal characteristics: age, educational level, ethnicity, parity;
5. Maternal characteristics: household income and marital status.

Due to multicollinearity, the three exposure variables could not be included in one regression model: MAD is the composite indicator of MDD and MMF. Therefore, we ran two models for each of the outcome variables. Firstly, we ran two models to determine the association between each of the IYCF indicators and URTI-model 1: To determine the association between MDD, MMF, and URTI; model 2: To determine the association between the composite indicator MAD and URTI. 

Secondly, we ran two models to determine the association between each of the IYCF indicators and LRTI-model 1: To determine the association between MDD, MMF, and LRTI; model 2: To determine the association between MAD and LRTI. Lastly, we ran two models to determine the association between each of the IYCF indicators and RTI-model 1: To determine the association between MDD, MMF, and RTI; model 2: To determine the association between the MAD and the LRTI.

Achieving the MDD, MMF and MAD indicators were coded as 1. Associations were presented as ORs with 95% confidence interval (CI) and *p*-values.

### 2.9. Ethical Considerations

The study received ethical approval from the Institutional Review Boards (IRB) of the Government of Suriname and Tulane University, New Orleans, LA, USA (VG 023-14). Written informed consent was obtained from the mothers of the cohort-enrolled children prior to participation. Assent was obtained from participants < 18 years of age. All participants were free to refrain from any part of the study at any time.

## 3. Results

### 3.1. Population Characteristics

**Figure 1:**
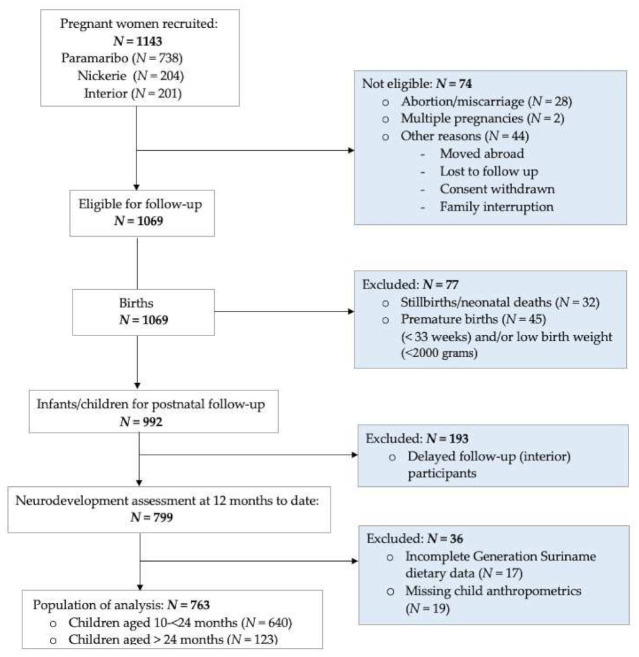
Flowchart with participant enrollment.

In total, 992 infants were eligible for postnatal follow-up. Due to delayed recruitment and data collection of the logistically difficult-to-reach participants in the Surinamese interior, a total of 799 Surinamese mother–child dyads were included in the current analysis (80.5%). Of those, 36 (4.5%) were excluded from analysis due to missing questionnaire data and child anthropometrics, yielding a sample size of 763 (Figure 1: Flowchart with participant enrollment). 

The mean ± SD age of the studied children was 18.13 ± 4.8 months. In terms of age group, 83.9% were in the age group < 24 months (younger), and 16.1% were ≥24 months of age (older).

Of all participants, 29.5% younger and 22.8% older children were reportedly being breastfed. The majority of enrolled mothers were residing in Paramaribo and Wanica (52.3%), 22.9% were of Hindustani descent. Over half (53.2%) had no, primary, or lower vocational or secondary education level, 65.0% had a household income below 3000 SRD. Further details on infant and maternal characteristics are displayed in Supplementary Tables S2 and S3.

### 3.2. Assessment of Respiratory Tract Infections 

#### 3.2.1. Upper or Lower Respiratory Tract Infections

Of all study children, 72.6% experienced at least one parent-reported RTI during the last six months before the 12-month follow-up. (Table 1). The number of children suffering from either an upper and/or lower RTI was significantly lower in the MDD- and MAD-achieved group, compared with the non-achieved group. (66.7% vs. 79.6%, 66.8% vs. 78.5%, *p* < 0.001, respectively).

#### 3.2.2. Upper Respiratory Tract Infections

Out of the 763 children, 71.7% experienced at least one parent-reported episode of an URTI in the six months before follow-up. Higher prevalences of URTI were observed in the groups of children not achieving either of the IYCF indicators. When the MDD and MAD indicators were achieved (65.4%), significantly lower prevalences of upper tract infections were found (*p* < 0.001). 

#### 3.2.3. Lower Respiratory Tract Infections

Approximately, one in eight (12.7%) children suffered from at least one LRTI as reported by the parent. Children that achieved the IYCF indicators were less likely to experience LRTI although this difference was not significant from either of the non-achieved groups (Table 1). 

### 3.3. Feeding Practices and Respiratory Tract Infections

The associations between IYCF practices and RTIs are displayed in Table 2 and Table 3. 

#### 3.3.1. IYCF Practices and Upper Respiratory Tract Infections 

Our hierarchical logistic regression models also confirmed that the IYCF indicators MDD and MAD remained inversely associated with (U)RTIs after adjusting for all covariates (Block 6); (OR 0.53; *p* < 0.001, and OR 0.55; *p* < 0.001, respectively). This strong association was also confirmed prior to adding the covariates (block 1). In addition to MDD and MAD, parity was identified as a predictor of RTIs (*p* = 0.03). Children of multiparous women had lower odds on (U)RTIs. (Table 2, Table 3 and Table A1).

#### 3.3.2. IYCF Practices and Lower Tract Infections

The Omnibus Test of Model Coefficients or our logistic regression output indicated that the null model was not outperformed, although the model was borderline significant. (Table 2 and Table 3: model 1: χ^2^ 23.99; df 16; *p* = 0.055 and model 2: χ^2^ 23.12; df 15; *p* = 0.056). Household income was found to be inversely associated with LRTIs. (See Table A1: model 1: OR 0.77; *p* < 0.02, model 2: OR 0.77; *p* < 0.03).

#### 3.3.3. Complementary Feeding Practices According to Place of Residence

##### Children < 24 Months of Age 

The highest proportion of younger children that met the MDD criteria resided in Nickerie. This proportion was significantly higher than in Paramaribo and the interior region (Table 4: 67.6%, 50.0%, and 36.5%, respectively, *p* < 0.001). No significant differences were found between Paramaribo and the interior region (See Table A2: multiple proportion test).

The number of children achieving appropriate meal frequency was significantly lower in the interior, compared with Paramaribo and Nickerie (60% vs. 94.2% and 93.0%, *p* < 0.001). With regard to the MAD, children residing in Nickerie had the highest probability to meet the requirements, compared with children in Paramaribo and the interior (62.7% vs. 47.8 and 29.6%, respectively; *p* < 0.001). 

##### Children ≥ 24 Months of Age 

Older children living in Nickerie were most likely to achieve the complementary feeding indicators. No significant differences were observed between the three regions with regard to achieving any of the three indicators.

## 4. Discussion

This study sought to (1) explore the association between respiratory tract infections and the WHO IYCF indicators among infants aged 10–33 months and (2) determine and compare the proportion of infants meeting the indicators in three differently populated regions in Suriname.

### 4.1. Association between Respiratory Tract Infections and IYCF Indicators Children (10–33) Months in Suriname 

Our findings suggest that MDD and MAD are significantly associated with upper respiratory tract infections. Even after the adjustment for covariates in our hierarchical regression model, the MDD and MAD indicators remained strongly associated with (upper) respiratory tract infections. These results offer powerful evidence of the importance of appropriate feeding practices in preventing (upper) respiratory tract infections. However, since this was a cross-sectional study, causality cannot be inferred from the results. Further prospective studies are needed to assess the MDD and MAD indicators as predictors of respiratory tract infections.

The direct comparison of our findings with other studies is difficult, as most studies investigated dietary patterns linked to respiratory diseases [28,29]. However, several studies have identified dietary diversity as a valid proxy indicator of micronutrient adequacy [30,31,32,33]. Lack of a diverse diet may deprive children of critical nutrients and therefore increase susceptibility to infections [34,35]. Our analyses support the compelling role of dietary diversity in (upper) respiratory tract infections. 

This study showed that in contrast to the MDD indicator, individually, the MMF indicator was not significantly associated with respiratory tract infections. This result was not fully in line with our expectations, since being fed insufficient quantities also can have detrimental effects on child health and development. The reasons for this non-significant finding in our study population are not completely understood, especially since meeting the minimum acceptable diet was strongly associated with lower odds of (upper) respiratory tract infections. A possible explanation could be that the MMF indicator only sets requirements for the minimum times a child should be fed and is unable to account for excessive frequencies of consuming foods. Recently, a prospective study hypothesized that high meal frequencies could potentially lead to significantly increased disease risk [36]. Thus, this would imply that consuming both insufficient and excessive quantities could potentially lead to an increased risk of respiratory infections. This could be a plausible explanation for finding the odds ratios close to 1 when assessing the association between minimum meal frequency and respiratory infections. 

As the minimum acceptable diet is the composite indicator of the meal frequency and dietary diversity, it captures the quantity and quality of nutrition [37,38]. The strong association between this indicator and respiratory tract infections was therefore not surprising. 

In addition to MDD and MAD, parity was identified as an independent factor associated with (upper) respiratory tract infections. Our results showed that the infants of multiparous mothers had lower odds of respiratory tract infections. Numerous studies suggest that compared with primiparous mothers, multiparous women have lower odds for preterm delivery and small-for-gestational-age offspring [39,40,41,42,43]. Additionally, the risk and severity of infectious diseases seem to increase with decreasing birth weight and gestational age [44,45,46]. Therefore, the odds of (upper) RTI could have indirectly been influenced by parity. 

Household income was negatively associated with the odds of lower respiratory tract infections. However, these results should be interpreted with caution since the Omnibus Test of Model Coefficients revealed that our model for lower respiratory tract infections was only borderline significant. This could be indicative of a poor fit [47]. A reasonable explanation for our borderline significance could be low statistical power, due to relatively low parent-reported lower respiratory tract infections. Therefore, the chance of detecting a true effect was reduced [48]. 

### 4.2. IYCF Indicators According to Place of Residence

There was a clear observation that our study participants under two, living in the remote tropical rainforest interior area, were less likely to achieve the feeding indicators. This is in concordance with the findings of the Suriname Multiple Indicator Cluster Survey (MICS-6). The survey additionally revealed the lowest literacy and wealth quintile index, as well as the highest percentage of age-appropriate breastfeeding and longest median duration of breastfeeding [20]. This is also consistent with the cross-sectional study of Getrouw et al., (2017), observing the highest proportion of breastfeeding initiation and duration among Maroon mothers [49]. 

Residents of the remote areas, “the food deserts,” are more vulnerable to food insecurities, compared with those living in the other regions. These food deserts are influenced by the lack of local supermarkets and grocery stores, and poor infrastructure—namely, the lack of access to public and personal transportation and the long distances between the residences and stores. Thus, poor socioeconomic status, inadequate food access, and low literacy among those in the interior negatively affect the complementary feeding practices but positively affect the duration and frequency of breastfeeding. 

### 4.3. Strengths and Limitations 

To our knowledge, this is the first study to ascertain the relationship between the WHO complementary IYCF indicators and respiratory tract infections among infants aged 10–33 months in Suriname. An additional strength of this study is that effort was made to determine child nutritional status and the proportion of infants meeting the indicators in three differently populated regions of Suriname: Paramaribo, Nickerie, and the tropical rainforest interior. 

However, some limitations exist in considering our findings. At the 12-month follow-up, the children’s age ranged from 10 to 33 months due to delayed recruitment and data collection of the logistically difficult-to-reach participants in the interior region. 

The WHO indicators were used to assess the feeding practices of all study children, including those above two years of age. However, as previously mentioned, we suggest our modifications were consistent with the WHO guidelines and were age appropriate. 

Due to the limited number of similar studies conducted in Suriname, the direct comparison of our findings with other studies could not be fully made. Therefore, some comparisons were made with studies conducted in other lower- and middle-income countries.

After excluding participants based on their birth weight and gestational age below 33 completed weeks, the sample size of our premature children was too small to include the covariate prematurity in our models, although it previously has been identified as a key variable influencing nutritional status and the incidence of respiratory tract infections. 

Another limitation of our study was that the models for lower respiratory tract infections, including covariates were a poor fit [47]. Thus, the data arising from these models should be interpreted with caution. Additionally, the explored independent predictors and potential confounders in this study may not be comprehensive, potentially limiting our understanding of the association between the nutrition indicators and respiratory tract infections. 

Data concerning the upper and lower respiratory tract infections were obtained by parent-reported physician visits, which could have led to misclassification bias. We acknowledge that a physician’s diagnosis would have been more accurate. However, the possibility of misclassification was considered by combining the upper and lower tract infections into one group. In addition, this study did not collect data on the number of episodes and the severity of the infections. 

Despite the widely accepted 24-h dietary recall method for dietary assessment, social desirability bias and recall bias from the mothers should be taken into consideration. Regarding child anthropometrics, measurement bias and inter-observer bias cannot be ruled out. We are aware that the WHO IYCF indicators were indeed designed to assess feeding practices in children aged 6–23 months; however, we imply that the modifications for the older children are age appropriate and consistent with the guidelines and previous studies. 

## 5. Conclusions

The WHO IYCF indicators MDD and MAD were found to be associated with at least one (upper) RTI. Thus, this study has shed more light on the clinical observation that adequate nutrition decreases the frequency and occurrence of RTI. Parity and household income were covariates significantly associated with (upper) respiratory tract infections. Suriname is a country with large socioeconomic disparities and limited effective public health interventions to improve child public health. Therefore, we recommend the implementation of culturally tailored large-scale nutrition-specific and nutrition-sensitive interventions to promote, protect, and support optimal and age-appropriate feeding practices, making the tropical rainforest interior a priority area. Additionally, further prospective studies are needed to elucidate the complex relationship between nutrition and respiratory tract infections exploring a larger set of potential confounding factors.

## Figures and Tables

**Table 1:** Associations between upper and/or lower respiratory tract infections, minimum dietary diversity (MDD), minimum meal frequency (MMF), and minimum acceptable diet (MAD).

Total (N = 763) Respiratory Tract Infections	Not Achieved MDD ^1^ (N = 367)	Achieved MDD (N = 393)	*p*-Value	Not Achieved MMF (N = 88)	Achieved MMF (N = 675)	*p*-Value	Not Achieved MAD ^1^ (N = 396)	Achieved MAD (N = 364)	*p*-Value
N (%)	N (%)	N (%)		N (%)	N (%)		N (%)	N (%)	
URTI 547 (71.7)	287 (78.2)	257 (65.4)	<0.001 *	64 (72.7)	483 (71.6)	0.82	306 (77.3)	238 (65.4)	<0.001 *
LRTI 97 (12.7)	50 (13.6)	45 (11.5)	0.37	13 (14.8)	84 (12.4)	0.54	52 (13.1)	43 (11.8)	0.58
RTI 554 (72.6)	292 (79.6)	262 (66.7)	<0.001 *	65 (73.9)	492 (72.9)	0.85	311 (78.5)	243 (66.8)	<0.001 *

^1^ Three missing, total N = 760. * Statistically significant. URTI: upper tract infection; LRTI: lower tract infection; RTI: respiratory tract infections.

**Table 2:** Hierarchical logistic regression model 1: Association between the WHO Infant and Young Child feeding (IYCF) indicators minimum dietary diversity (MDD), minimum meal frequency (MMF), and respiratory tract infections (RTIs).

Predictors	URTI			LRTI			RTI		
	β	OR	95% CI	β	OR	95% CI	β	OR	95% CI
(Constant)	2.61	13.64		−3.28	0.04		2.61	13.60	
Model 1									
MDD	−0.62	0.53	0.38, 0.76 *	−0.15	0.86	0.53,1.37	−0.65	0.52	0.37, 0.74 *
MMF	0.05	0.95	0.53, 1.71	0.28	1.33	0.60, 2.91	−0.02	0.98	0.54, 1.77
Cumulatively adjusted for									
currently breastfed (0 = not breastfed)	−0.13	0.88	0.59, 1.31	0.04	1.04	0.61, 1.78	−0.15	86	0.58, 1.29
Cumulatively adjusted for									
birth weight (in grams)	0.00	1.00	0.99, 1.00	0.00	1.00	1.0, 1.0	0.00	1.00	0.99, 1.00
gender (0 = female)	0.13	1.13	0.81, 1.59	0.21	1.23	0.77, 1.96	0.15	1.16	0.83, 1.63
Cumulatively adjusted for									
food allergies (0 = no)	0.23	1.26	0.55, 2.89	0.45	1.57	0.60, 4.08	0.14	1.15	0.49, 2.66
Cumulatively adjusted for									
Maternale age (in years)	−0.01	0.99	0.96, 1.02	0.03	1.03	0.99, 1.07	−0.01	0.99	0.96, 1.02
Educational level	0.09	1.09	0.87, 1.36	0.02	1.03	0.75, 1.39	0.12	1.12	0.89, 1.40
(ref = no or primary level)									
Parity (0 = primi)	−0.45	0.64	0.43, 0.96 *	0.08	1.08	0.61, 1.89	−0.43	0.65	0.43, 0.98 *
Maternal ethnicity (ref = mixed)									
(Creole)	0.12	1.13	0.62, 2.06	0.34	1.4	0.60, 3.27	0.11	1.11	0.60, 2.06
(Hindustani)	0.013	1.01	0.75, 1.80	−0.09	0.92	0.38, 2.20	−0.08	0.92	0.51, 1.65
(Indigenous)	0.181	1.20	0.58, 2.49	0.59	1.80	0.68, 4.75	0.19	1.21	0.57, 2.56
(Javanese)	0.01	1.01	0.50, 2.05	0.23	1.26	0.46, 3.42	−0.04	0.96	0.47, 1.97
(Tribal)	0.03	1.04	0.55, 1.95	−0.66	0.52	0.19, 1.39	−0.06	0.94	0.49, 1.79
Cumulatively adjusted for Marital status									
(ref = not married/living with partner)	−0.28	0.76	0.43, 1.34	0.18	0.1.20	0.55, 2.60	−0.25	0.78	0.44, 1.34
Household income (in SRD)	−0.05	0.95	0.81, 1.11	−0.26	0.77	0.62, 0.96 *	−0.08	0.92	0.79, 1.08

OR: Odds ratio; 95% CI: confidence interval; SRD: Surinamese Dollars. Model 1 (including MDD and MMF) was not significant for lower respiratory tract infections (χ^2^; 23.99, df; 16, *p* = 0.056, R^2^ Nagelkerke; 0.09). * Significant.

**Table 3:** Hierarchical logistic regression model 2: Association between the WHO Infant and Young Child feeding (IYCF) indicator minimum acceptable diet (MAD) and respiratory tract infections (RTIs).

Predictors	URTI			LRTI			RTI		
	β	OR	95 % CI	β	OR	95% CI	β	OR	95% CI
(Constant)	2.60	13.40		−3.07	0.05		2.61	13.60	
Model 2									
MAD	−0.59	0.55	0.39, 0.78 *	0.02	0.86	0.53,1.37	−0.60	0.55	0.37, 0.74 *
Adjusted for currently breastfed	−0.16	0.85	0.58, 1.25	0.01	1.01	0.60, 1.72	−0.19	83	0.58, 1.29
(0 = not breastfed)									
Cumulatively adjusted for									
birth weight (in grams)	0.00	1.00	0.99, 1.00	0.00	1.00	1.0, 1.0	0.00	1.00	0.99, 1.00
gender (0 = female)	0.11	1.11	0.80, 1.57	0.20	1.23	0.77, 1.95	0.14	1.15	0.81, 1.61
Cumulatively adjusted for									
food allergies (0 = no)	0.20	1.22	0.53, 2.81	0.44	1.55	0.60, 4.04	0.11	1.12	0.49, 2.57
Cumulatively adjusted for									
Maternale age (in years)	−0.01	0.99	0.96, 1.02	0.02	1.03	0.99, 1.07	−0.01	0.99	0.96, 1.02
Educational level	0.09	1.09	0.88, 1.36	0.03	1.03	0.76, 1.40	0.12	1.12	0.90, 1.40
(ref = no or primary level)									
Parity (0 = primi)	−0.46	0.63	0.42, 0.94 *	0.07	1.07	0.61, 1.89	−0.45	0.64	0.43, 0.96 *
Maternal ethnicity (ref = mixed)									
(Creole)	0.15	1.16	0.64, 2.12	0.37	1.4	0.62, 3.36	0.14	1.15	0.63, 2.12
(Hindustani)	0.03	1.03	0.58, 1.84	−0.08	0.92	0.38, 2.20	−0.06	0.94	0.53, 1.69
(Indigenous)	0.19	1.21	0.59, 2.52	0.55	1.80	0.66, 4.58	0.20	1.22	0.58, 2.58
(Javanese)	0.02	1.02	0.50, 2.07	0.23	1.26	0.46, 3.43	−0.03	0.97	0.47, 1.99
(Tribal)	0.07	1.08	0.58, 2.02	−0.66	0.52	0.19, 1.39	−0.02	0.98	0.52, 1.86
Marital status	−0.29	0.75	0.42, 1.32	0.18	0.1.20	0.55, 2.59	−0.26	0.77	0.44, 1.38
(ref = not married/living with partner)									
Household income (in SRD)	−0.04	0.96	0.81, 1.12	−0.25	0.77	0.62, 0.96 *	−0.07	0.93	0.79, 1.08

OR: Odds ratio; 95% CI: confidence interval; SRD: Surinamese Dollars. Model 2 was not significant for lower respiratory tract infections (χ^2^; 23.12, df; 15, *p* = 0.056, R^2^ Nagelkerke; 0.08). * Significant.

**Table 4:** Minimum dietary diversity (MDD), minimum meal frequency (MMF), and minimum acceptable diet (MAD) according to the place of residence.

		Place of Residence					Place of Residence				
Total (N = 586)	Overall Practice (95% CI)	<24 Months (N = 481)	P (N = 224)	N (N = 142)	I (N = 115)	*p*-Value	≥24 Months (N = 105)	P (N = 59)	N (N = 30)	I (N = 16)	*p*-Value
N (%)	N (%)	N (%)	N (%)	N (%)	N (%)		N (%)	N (%)	N (%)	N (%)	
Achieved MDD ^1^ N = 315 (53.7)	(50.0–58.0)	250 (52.3)	112 (50.0)	96 (67.6)	42 (36.5)	<0.001 *	65 (61.9)	33 (55.9)	23 (76.7)	9 (56.3)	0.14
Achieved MMF N = 503 (85.8)	(82.8–88.4)	412 (87.7)	211 (94.2)	132 (93.0)	69 (60.0)	<0.001 *	91 (86.7)	52 (88.1)	27 (90.0)	2 (75.0)	0.32
Achieved MAD ^1^ N = 289 (49.3)	(45.5–53.6)	230 (47.8)	107 (47.8)	89 (62.7)	34 (29.6)	<0.001 *	59 (56.2)	31 (52.5)	20 (66.7)	8 (50.0)	0.39

^1^ Three missing, total N = 583; N = number of infants meeting the WHO IYCF indicators; () = percentage of infants achieving the WHO IYCF indicators according to the place of residence; P: Paramaribo, N: Nickerie, I: Tropical rainforest interior: Marowijne, Brokopondo, Sipaliwini. * statistically significant.

## Data Availability

The data presented in this study can be made available based on a reasonable request. Such requests should be directed to the PI’s of the Cari bean Consortium for Research in environmental and Occupational Health (CCREOH) through the intermediary of the corresponding author.

## Supplementary Materials

The following are available online at https://www.mdpi.com/article/10.3390/ijerph182010739/s1, Table S1: Modified versions WHO core IYCF indicators concerning complementary feeding practices, Table S2: Distribution of infant characteristics at year 1 follow-up, Table S3: Distribution of maternal characteristics at year 1 follow-up.

## Appendix A

**Table A1:** Predictors of (upper and lower) respiratory tract infections.

Predictors	Upper RTI			* Lower RTI			Upper or Lower RTI		
	OR	95% CI	*p*	OR	95% CI	*p*	OR	95% CI	*p*
Model 1:MDD/MMF MDD Parity Household income	0.53 0.64	[0.38, 0.76] [0.43, 0.96]	<0.001 0.03	0.77	[0.62,0.96]	0.02	0.51 0.65	[0.36, 0.72] [0.43, 0.98]	<0.001 0.04
Model 2: MAD MAD Parity Household income	0.55 0.63	[0.39, 0.78] [0.42, 0.94]	<0.001 0.03	0.77	[0.62, 0.97]	0.03	0.55 0.64	[0.39, 0.77] [0.43, 0.96]	<0.001 0.03

* Model 1 and 2 were borderline significant for lower tract infections.

## Appendix B

**Table A2:** * Multiple proportion test to compare the proportion of children meeting the WHO IYCF indicators across Paramaribo, Nickerie, and the tropical rainforest interior.

	Place of Residence	P ^1^	N ^2^	I ^3^
IYCF Indicators				
Achieved MDD			P I	
Achieved MMF		I	I	
Achieved MAD		I	P I	

* Significance was determined at the *p* < 0.05 level, two sided. ^1^ Paramaribo, ^2^ Nickerie, ^3^ Interior.
