# Supplementary material for: Poor Adherence to the WHO Guidelines on Feeding Practices Increases the Risk for Respiratory Infections in Surinamese Preschool Children
Source: Int J Environ Res Public Health. 2021 Oct 13;18(20):10739. doi: 10.3390/ijerph182010739 (PMC8536009; doi:10.3390/ijerph182010739)
Supplement: Supplementary file 1 [file ijerph-18-10739-s001.zip › ijerph-1314955-supplementary.pdf]

Supplementary materials

Table S1: Modified versions WHO core IYCF indicators concerning complementary feeding practices [19,23]

	**Minimum dietary diversity (MDD)**
**Definition:**	Proportion of children aged 6–23 months, who received foods from at least four out of seven food groups the previous day.<br><br>The seven foods groups of this indicator were categorized as follows:<br>1. grains, roots and tubers<br>2. legumes and nuts<br>3. dairy products (milk, yogurt, cheese)<br>4. flesh foods (meat, fish, poultry and liver/organ meats)<br>5. eggs<br>6. vitamin-A rich fruits and vegetables<br>7. other fruits and vegetables<br>Modification:<br>Group 6: Vitamin-A rich fruits and vegetables and group 7: non-vitamin-A rich fruits and vegetables, were combined in one food group. The MDD was considered achieved as per the WHO guidelines[24]
**Calculation:**	Children 6–23 months of age who received foods from ≥4 food groups during the previous day / Children 6–23 months of age
	**Minimum meal frequency (MMF)**
**Definition:**	Proportion of breastfed and non-breastfed children 6–23 months of age who receive solid, semi-solid, or soft foods (including milk feeds for non-breastfed children) the minimum number of times or more the previous day.<br>Minimum is defined as:<br>— 3 times for breastfed children 9–23 months<br>— 4 times for non-breastfed children 6–23 months<br><br>Modification:<br>Minimum is defined as:<br>— 4 times per day for breastfed children aged ≥24 months<br>— 5 times (three meals with two additional snacks) per day for non-breastfed children aged ≥24 months[25]<br><br>Remarks:<br>*For breastfed children, this indicator assumes average breast milk intake. Therefore, the meal frequency indicator was calculated without including milk feeds.
**Calculation:**	Breastfed children 6–23 months of age who received solid, semi-solid or soft foods the minimum number of times or more during the previous day / Breastfed children 6–23 months of age<br><br>and<br><br>Non-breastfed children 6–23 months of age who received solid, semi-solid or soft foods the minimum number of times or more during the previous day

	Non-breastfed children 6–23 months of age
	**Minimum acceptable diet (MAD)**
**Definition:**	Proportion of children 6–23 months of age who receive a minimum acceptable diet (apart from breast milk).<br>The MAD is a composite indicator of the MDD and MMF.<br><br><u>Remarks:</u><br>To calculate this indicator for the non-breastfed children, milk feeds were excluded from the dietary diversity score to avoid double-counting.<br>— Breastfed children who met both the MDD and the MMF were considered to have achieved the required MAD.<br>— Non-breastfed children were considered to have achieved the required MAD if they received at least 2 milk feedings and met the MDD (excluding milk feeds) and the MMF during the previous day.<br><br><u>Modification:</u><br>Children aged ≥ 24 months achieved the MAD if MDD and MMF were met[24].
**Calculation:**	Breastfed children 6–23 months of age who had at least the MDD and MMF during the previous day<br>―――――――――――――<br>Breastfed children 6–23 months of age<br><br>and<br><br>Non-breastfed children 6–23 months of age who had at least 2 milk feedings and had at least the MDD not including milk feeds and the MMF during the previous day<br>―――――――――――――<br>Non-breastfed children 6–23 months of age

Table S2: Distribution of infant characteristics at year 1 follow-up

	**Total**		**Children aged <24 months** (<730 days of age)		**Children aged ≥24 months** (≥730 days)		
	N = 763 (100%)		*N*= 640 (83.9%)		*N*= 123 (16.1%)		
**Infant characteristics**	***N***	**%**	***N***	**%**	***N***	**%**	***p*-value**
**Gender**							.62
Male	394	51.6	333	52	61	49.6	
Female	369	48.4	307	48	62	50.4	
Missing			0	0.0	0	0.0	
**Gestational age**							0.04*
Moderately preterm (33 + 0 - 36 + 6 weeks)	28	3.7	19	3.0	9	7.3	
Term births (37 + 0 weeks)	687	90	576	90.0	111	90.2	
Missing	48	6.3	45	7.0	3	2.4	
**Currently breastfed**							.13
Yes	217	28.4	189	29.5	28	22.8	
No	546	71.6	451	70.5	95	77.2	
Missing	0	0.0	0	0.0	0	0.0	
**Food allergy**							0.72
Yes	36	4.7	31	4.8	5	4.1	
No	725	95	608	95.0	117	95.1	
Missing	2	0.3	1	0.2	1	0.8	
	**[1]M**	**[2]SD**	**[1]M**	**[2]SD**	**[1]M**	**[2]SD**	
**Age at first follow-up (months)**	18.13	4.81	16.41	2.80	27.06	2.67	<.001*

Table S3: Distribution of maternal characteristics at year 1 follow-up

**Distribution of maternal characteristics**	**Total**		**Children aged <24 months (<730 days)**			**≥ Children aged 24 months (≥730 days)**		
	N = 763 (100%)		*N*= 640 (83.9%)			*N* = 123 (16.1%)		
**Maternal characteristics**	***N***	**%**	*N*	%		*N*	%	***p*-value**
**Maternal age at intake**								0.44
16–19 years	84	11.0	67	10.5		17	13.8	
20–24 years	172	22.5	149	23.3		23	18.7	
25–29 years	213	27.9	180	28.1		33	26.8	
30–34 years	181	23.7	150	23.4		31	25.2	
35–39 years	87	11.4	75	11.7		12	9.8	
40+ years	26	3.4	19	3.0		7	5.7	
Missing	0	0.0	0	0.0		0	0.0	
**Parity**								0.11
Primiparous	254	33.3	205	32.0		49	39.8	
Multiparous	501	65.7	427	66.7		74	60.2	
Missing	8	1.0	8	1.3		0	0	
**Ethnicity**								0.27
Creole	166	21.8	144	22.5		22	17.9	
Hindustani	175	22.9	142	22.2		33	26.8	
Indigenous	90	11.8	75	11.7		15	12.2	
Javanese	70	9.2	61	9.5		9	7.3	
Maroon	163	21.4	141	22.0		22	17.9	
Mixed	92	12.1	71	11.1		21	17.1	
Missing	7	0.9	6	0.9		1	0.8	
**Marital status**								0.44
Married or living with partner	667	87.4	557	87%		110	89.4%	
Not married/not living with partner	90	11.8	78	12.2%		12	9.8%	
Missing	6	0.8	5	0.8%		1	0.8%	
**Educational level**								0.29
No or primary level	165	21.6	143	22.3		22	17.9	
Lower vocational/secondary	241	31.6	200	31.3		41	33.3	
Upper vocational/secondary	226	29.6	182	28.4		44	35.8	
Tertiary	126	16.5	110	17.2		16	13.0	

Missing	5	0.7	5	0.8		0	0.0	
**Household income SRD[3]**								0.37
<400	31	4.1	28	4.4		3	2.4	
400–799	89	11.7	75	11.7		14	11.4	
800–1499	134	17.6	110	17.2		24	19.5	
1500–2999	223	29.2	185	28.9		38	30.9	
3000–4999	171	22.4	142	22.2		29	23.6	
5000–9999	64	8.4	49	7.7		15	12.2	
10000–14999	18	2.4	18	2.8		0	0.0	
15000+	4	0.5	4	0.6		0	0.0	
Missing	29	3.8	29	4.5		0	0.0	
**Community factors:**								
**Place of residence**								0.39
- Paramaribo, Wanica	399	52.3	330	51.6		69	56.1	
- Commewijne, Saramacca, Coronie, Nickerie, Para	229	30.0	191	29.8		38	30.9	
- Tropical rainforest interior: Marowijne, Brokopondo, Sipaliwini	131	17.2	115	18.0		16	13.0	
Missing	4	0.5	4	0.6		0	0.0	
**Access to health care**								
**First antenatal care visit**								0.48
First trimester	491	64.4	404	63.1		87	70.7	
Second trimester	211	27.7	181	28.3		30	24.4	
Third trimester	7	0.9	6	0.9		1	0.8	
Missing	54	7.1	49	7.7		5	4.1	
**Health Insurance**								0.13
Insured	498	65.3	401	62.7		97	78.9	
Not insured	37	4.8	26	4.1		11	8.9	
Missing	228	29.9	213	33.3		15	12.2	

[1,2]Data are presented as mean (M) with standard deviation (SD) or N %

[3] Srd = Surinamese dollar, equivalent to 0.07 USD

* statistically significant
